# Development of a model care pathway for the management of Hymenoptera venom allergy: evidence-based key interventions and indicators

**DOI:** 10.1186/s13601-020-00312-3

**Published:** 2020-03-04

**Authors:** Maria Beatrice Bilò, Alice Corsi, Valerio Pravettoni, Donatella Bignardi, Patrizia Bonadonna, Oliviero Quercia, Marina Mauro, Elio Novembre, Rebecca Micheletti, Roberto Papa

**Affiliations:** 1 Allergy Unit, Department of Clinical and Molecular Sciences, Università Politecnica delle Marche, Ancona, Italy; 2 Department of Internal Medicine, University Hospital Ospedali Riuniti di Ancona, Ancona, Italy; 3 Postgraduate School of Allergy and Clinical Immunology, Università Politecnica delle Marche, Ancona, Italy; 4 U.O.C. General Medicine-Immunology and Allergology, Foundation IRCCS Ca’ Granda Ospedale Maggiore Policlinico, Milan, Italy; 5 U.O.C. Allergology, Ospedale Policlinico San Martino, Genoa, Italy; 6 U.S.D. Allergology Integrated University-Hospital of Verona, Verona, Italy; 7 High Specialization Unit of Allergology, Hospital of Faenza, AUSL (Local Health Unit) of Romagna, Romagna, Italy; 8 U.O.S. Allergology ASST Lariana, Como, Italy; 9 Complex Organizational Unit of Allergology, University-Hospital A. Meyer, Florence, Italy; 10 Postgraduate School of Hygiene and Preventive Medicine and Public Health, Università Politecnica delle Marche, Ancona, Italy; 11 S.O. Hospital Medical Management, University Hospital Ospedali Riuniti di Ancona, Ancona, Italy

**Keywords:** Care pathway, Clinical pathway, Core activity, Flow diagram, Hymenoptera venom allergy, Key interventions, Quality indicators

## Abstract

**Background:**

Hymenoptera venom allergy (HVA) is an underestimated condition representing an important cause of morbidity and mortality worldwide. Preventing future allergic reactions in patients who have already developed a systemic reaction is based on the correct management of the acute phase of the reaction followed by a correct diagnosis and, where indicated, prescription of adrenaline autoinjectors and VIT. A possible strategy to optimize care processes and to improve outcomes is the implementation of a Diagnostic and Therapeutic Care Pathways, also known as Integrated Care Pathways or Clinical Pathways (CPWs). The aim of the care pathway is to enhance the quality of care by improving risk‐adjusted patient outcomes, promoting patient safety, increasing patient satisfaction, and optimizing the use of resources. To our knowledge, currently in Italy as well as in Europe, there is no CPWs codified for the management of HVA patients. This paper describes the development of the clinical content of a care pathway for the management of HVA.

**Methods:**

The methodology applied is based on the eight step method to build the clinical content of an evidence-based care pathway suggested by Lodewijckx et al.

**Results:**

Three hundred and seventeen different clinical activities were extracted from the selected literature. The expert panel was involved in their evaluation, expressing a judgment of relevance through the Delphi study. As a result, 126 clinical activities were appraised to be valid and feasible. The final recommendations (126) were translated into 123 key interventions. Six indicators were produced by the clinical activities.

**Conclusion:**

A set of 123 key interventions and of six process indicators were found to be appropriate for the development and standardization of the clinical content of the Hymenoptera venom allergy care pathway.

## Background

Hymenoptera venom allergy (HVA) is an underestimated condition representing an important cause of morbidity and mortality worldwide. It can occur with varying degrees of severity (from local to anaphylactic reactions) and can sometimes be fatal. According to the data from the European Anaphylaxis Registry, HVA is the first cause of severe reactions in adults and the second in children (48.2% and 20.2% respectively) [1]. In different countries, HVA is responsible for about 20% of the total cases of fatal anaphylaxis [2]. Overall, the incidence of mortality in the various European countries is between 0.03/million/year in Italy and 0.48 in France. However, mortality data are generally underestimated, as deaths are likely to be attributed by mistake to other causes, in particular to cardiac disorders. Moreover, up to 8% of adult patients with HVA may simultaneously suffer from a systemic mastocytosis (SM). SM is a clonal mast cell (MC) disease that can lead to potentially fatal anaphylactic reactions caused by excessive MC mediator release. Anaphylaxis is the most severe clinical manifestation of SM and is characterized by hypotension and syncope in the absence of urticarial and angioedema [3].

Hymenoptera venom allergy diagnosis is based on clinical history and results of in vivo and in vitro tests (mainly, specific IgE detection) [4]. Skin tests are the gold standard for diagnosis and should be carried out at least 2 weeks after the last sting, to exclude a false negative response during the refractory period. The presence of serum specific IgE to Hymenoptera venoms can be detected immediately after sting, even if the best period for their determination is 1–4 weeks after the sting. Diagnosis could be complicated by sensitization to multiple venoms (*Apis mellifera*/vespids or *Vespula* spp./*Polistes dominula*) in patients who have not identified the stinging Hymenoptera. The availability on the market of some major allergens expressed in recombinant form allows evaluating the specific IgE to individual molecular markers (CRD, Component-Resolved-Diagnosis). CRD may discriminate between primary sensitization and cross-reactivity in patients with double and multiple positivity of diagnostic tests with whole extracts [5].

As for therapy, adrenaline is the treatment of choice for acute anaphylaxis; it slows the progression of symptoms and can prevent the development of fatal or biphasic reactions [6]. All patients with a history of an anaphylactic reaction should be provided with an adrenaline autoinjector [7]. Venom immunotherapy (VIT) is the most effective treatment for subjects who developed a systemic allergic reaction (SR) after Hymenoptera sting, since currently it is the only treatment able to effectively prevent SR in case of a re-sting even after its discontinuation [8].

Preventing future allergic reactions in patients who have already developed a SR is based on the correct management of the acute phase of the reaction followed by a correct diagnosis and, where indicated, prescription of an adrenaline autoinjector and VIT.

A possible strategy to optimize care processes and to improve outcomes is the implementation of Diagnostic and Therapeutic Care Pathways, also known as Integrated Care Pathways or Clinical Pathways (CPWs). CPWs are complex interventions for mutual decision-making, organization and standardization of predictable care for a well-defined group of patients during a well-defined period [9].

The aim of the care pathway is to enhance the quality of care by improving risk‐adjusted patient outcomes, promoting patient safety, increasing patient satisfaction, and optimizing the use of resources [9]. Clinical pathways are primarily considered to be tools for designing care processes, implementing clinical governance, streamlining delivered care, improving the quality of clinical care and ensuring that clinical care is based on the latest research.

Standardization of the clinical care process through integration of evidence-based knowledge has proven to be an effective strategy for reducing unwanted variations in treatment, minimizing the probability of medical errors and improve the quality of the healthcare [10, 11]. The reduction of variability is in fact the key to quality.

Evidence-based clinical pathways can be effective tools for organizing evidence into multidisciplinary care plans for local work processes and can be a model for making evidence more actionable for providers at the point of care [12]. To our knowledge, currently in Italy as well as in Europe, there is no CPWs codified for the management of HVA patients.

This paper describes the development of the clinical content of a care pathway for the management of HVA.

## Methods

The methodology applied is based on the eight-step method to build the clinical content of an evidence-based care pathway as suggested by Lodewijckx et al. [10]. The steps applied for the development of the model pathway are shown below.

### Selection of an expert panel

A national expert panel was involved in each step of the development of the model pathway, in order to guarantee the clinical validity and practicability of the path.

The expert panel consisted of seven doctors with clinical and scientific expertise in HVA, two doctors with clinical and scientific expertise in emergency medicine and two pharmacists.

Moreover, the expert panel was supported by a technical-methodological coordination group made of one medical doctor, with scientific expertise in development and implementation of care pathways, one medical allergist from the expert panel and two post-graduate medical doctors (one post-graduate medical doctor in Allergy and Clinical Immunology and another one in Hygiene and Preventive Medicine and Public Health).

A first face-to-face meeting of the panel members was conducted in July 2018 to choose the protocol for search, evaluation and synthesis of best evidence for the realization of the CPW.

### Literature search and selection of recommendations

To identify the reference guidelines (LGs) an extensive literature review was conducted from July 2018 up to September 2018, with the aim of identifying the good practices to be included in the document.

The following resources were explored: (i) websites of European Academy of Allergy and Clinical Immunology (https://www.eaaci.org/); (ii) websites of The American Academy of Allergy, Asthma and Immunology (https://www.aaaai.org/); (iii) National Institute for Health and Clinical Excellence (NICE) (www.nice.org.uk); (iv) electronic database (Medline).

For Medline, the following terms were used: “guidelines” combined with “anaphylaxis”, “adrenaline” “Hymenoptera venom allergy”, “venom immunotherapy”, and “Systemic mastocytosis” combined with “Hymenoptera venom allergy”, “Allergy”, “Anaphylaxis”. All synonyms for these terms were included in the search.

Participants were given the opportunity to provide any additional recommendations from other good quality supplementary documents (Position Paper and Review) or, alternatively, to produce specific recommendations indicated as Good Practice Point (GPP), in the absence of evidence of effectiveness in the selected LGs.

The selected literature was thoroughly screened for identification of all possible recommendations by the members of the technical-methodological coordination group. The recommendations were extracted and listed both in English and Italian languages, and the corresponding literature sources were recorded.

### Delphi consensus method

The Delphi method was used in order to select relevant statements [13].

Two rounds were used to reach consensus. The first Delphi survey was conducted through email between October 2018–November 2018. In the first round, the panelists rated the relevance of each recommendations on a scale of 1 to 9, with 1 being certainly irrelevant and 9 being certainly relevant. They have also had the chance to record comments to explain their scores.

Completed score-sheets with any comments were then returned by email to the technical-methodological coordination group. The scores and comments were recorded onto a central database file. Feedback on the round one responses was provided to all panelists through central tendencies (median, mode) of each statement.

All panel members attended the second round face-to-face meeting, which occurred in December 2018, to discuss the results of the first round. The same analysis procedure was applied to the second round rating during the meeting. Consensus was defined as agreement by at least 75% of the panel members. All the statements that were rated 8 or 9 by at least 75% of the panelists in round two have been included in the final clinical pathway.

### Selection of key interventions

One of the active ingredients in care pathways is the integration of a set of evidence-based key interventions (KI) that may assist clinicians in selecting the best treatment options and in delivering safe and effective care.

Therefore, the selected recommendations were translated into key interventions. Each key intervention has been included a detailed description (rationale), which addresses the reason why the key intervention is performed (illustrating the expected impact on patient outcomes) and a description of the intervention (core activity), as a set of good quality tests, which define the exact content of the key intervention to be guaranteed to the patient.

The core activities allow contextualization of specific operating activities in greater details and meet a series of criteria: (i) core activities almost always require to be carried out by a specific member of the clinical care team (doctor, nurse, pharmacist etc.); (ii) core activities need to be carried out at a specific point in the treatment pathway or at a specific stage.

### Translation into a set of clinical indicators

To study the quality of care and the impact of care pathways appropriately, a valid and feasible set of process and outcome indicators needs to be defined. Therefore, besides the set of key interventions, it was necessary to develop a set of process and outcome indicators to verify compliance to key interventions and to follow up the impact on outcomes.

Clinical indicators are measures of clinical care which may, when assessed over time, provide a method of assessing the quality and safety of care. They are measures of the process or outcomes of patient care and they are used by health systems and services to identify areas of concern which might require further review or development. They are potentially important tool designed to help clinicians and healthcare organizations to assess the quality of care being provided against agreed evidence bases recommendations [14].

The key interventions that had obtained an average score higher than 8.8 were translated into a set of clinical indicators, which are relevant to study quality of care and impact of care pathways in HVA patients. The reason for choosing this assessment threshold is that identifying one or more indicators for each of the selected recommendations would have led to the production of a large number of indicators and standards difficult to measure. The group was given the opportunity to integrate the list with further adequately motivated performance indicators.

According to the commonly used Donabedian Model, quality of healthcare can be measured by three types of indicators: structure, process and outcome indicators [14]. In the HVA CPW only process indicators have been identified. The process indicator measures the appropriateness and completeness of information obtained through clinical history, physical examination, diagnostic tests; justification of diagnosis and therapy; technical competence in the performance of diagnostic and therapeutic procedures, evidence of preventive management in health and illness; coordination and continuity of care and therapy [15].

### Flow-diagram development

Finally, a process flow diagram was developed. CPWs can be viewed as algorithms as much as they offer a flow chart format of the decisions to be made and the care to be provided for a given patient or patient group for a given condition in a step-wise sequence; these detail the recommended algorithm of actions and level of care. The hierarchical algorithm is a snapshot of the process at its highest level; it focuses the user’s attention on the main decision nodes. It provides a mental model for the multidisciplinary team and helps to manage complexity [15].

## Results

Seventeen publications, among which six were international guidelines, were identified from literature search, which were screened for identification of all possible statements/recommendations [3, 6–8, 16–28].

There was no need to review any evidence with a GPP judgment from the panel of experts.

Three hundred and seventeen different recommendations were extracted from the selected literature. The expert panel was involved in their evaluation, expressing a judgment of relevance through the Delphi method. One hundred twelve recommendations had score (median, mode) higher than eight, one hundred ten recommendations had reached the agreement of at least 75% of the panel and were including in the CPW. Out of the remaining 205 recommendations, 202 had score between 4 and 7.9 and three had score lower than 4. The participants received a report, which illustrated the results of the first evaluation for each recommendation. A subsequent consensus meeting was held with the entire expert panel in order to discuss in the areas of disagreement and other two recommendations (out of 112) with score (median, mode) higher than eight that had not reached the agreement at 75% and recommendations with 4–7.9 score to make a final selection. The discussion then focused on the areas of disagreement that emerged. Consensus was reached for 16 of 204 recommendations.

As a result, 126 recommendations were appraised to be valid and feasible. They were categorized into five groups: 10 statements on emergency treatment of patients with HVA (ET), 27 statements on diagnosis of HVA (D), 26 statements on pharmacological therapy (PT), 47 statements on immunotherapy (VIT) and finally 16 on mastocytosis and HVA (M).

**Fig. 1:**
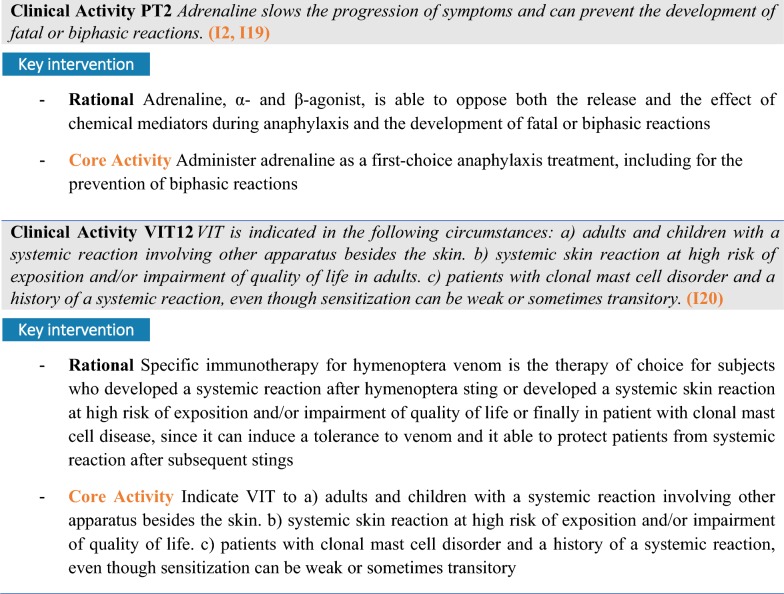
Examples of a key intervention. *PT* pharmacological therapy, *VIT* venom immunotherapy

The final recommendations (126) were then translated into 123 key interventions (similar recommendations have been combined into a single key intervention). An example of a key intervention (rational and core activity) is provided in Fig. 1. All key interventions are listed in Table 1.

**Table 1:** Selected key interventions as product of recommendations

Sub group	Refs.	Original recommendation	Key intervention (core activity)	Link to flow-chart
Emergency department/allergy specialist (ES)				
ES1	[19]	Document the acute clinical features of the suspected anaphylactic reaction (rapidly developing, life-threatening problems involving the airway [pharyngeal or laryngeal edema] and/or breathing [bronchospasm with tachypnea] and/or circulation [hypotension and/or tachycardia] and, in most cases, associated skin and mucosal changes)	Provide clinical symptoms of the acute phase in case of a suspected anaphylactic reaction	I1
ES2	[16]	Base the diagnosis of anaphylaxis on the history and physical examination, using scenarios described by the National Institutes of Allergy and Infectious Disease (NIAID) Panel but recognizing that there is a broad spectrum of anaphylaxis presentations that require clinical judgment. Do not rely on signs of shock for the diagnosis of anaphylaxis	Diagnose anaphylaxis using scenarios described by the NIAID panel, and do not rely only on shock signs	I12, I13
ES3	[7]	For diagnosis of doubtful reactions, collect blood (ideally within 1–2 h but no later than 4 h from the onset of symptoms) for serum tryptase testing (3 ml clotted sample, serum separated and froze)	Dose serum tryptase, within 4 h, in case of a doubtful reaction	I3
ES4	[7]	Record the daily medication and any additional self-medication taken on the day of the sting, in particular ACE inhibitor or beta-blocker	Record drug therapy assumed on the day of the sting, paying close attention to cardiological and/or hypotensive therapies	I2
ES5	[28]	Glucagon sometimes needed in patients taking a beta-adrenergic blocker who have hypotension and bradycardia and who do not optimally respond to adrenaline. Glucagon can be administered in adult patients intravenously at a dose of 1 mg as an initial IV bolus, repeatable every 5 min, increasing the dosage to 3–5 mg if necessary. The administration for continuous infusion in syringe pump should be carried out at the dose of 1–5 mg/h	Administer glucagone in patients under beta-blocking therapy with hypotension and low heart rate when they do not react optimally to adrenaline administration, taking into consideration it is an “off-label” therapy	I2
ES6	[28]	The patient, after receiving the appropriate therapies and obtained the resolution of clinical picture, should be kept under observation and monitored for at least 6–8 h up to 24 h depending on severity and characteristics of the reaction at onset, comorbidity and risk factors	Observe and monitor the patient after clinical resolution for at least 6–8 h and up to 24 h depending on the patient’s clinical conditions	I4, I5, I6, I7
ES7	[7]	After emergency treatment for suspected ISA, prescribe the patient (or, as appropriate, their parent and/or caregivers) an AAI that is appropriate for age and body mass. Patients must receive a referral to a specialist allergy service	Prescribe an adrenaline auto-injector after emergency treatment of the insect sting anaphylaxis	I10, I19
ES8	[19]	Before discharge a healthcare professional with the appropriate skills and competencies should offer people (or, as appropriate, their parent and/or carer) the following: Information about anaphylaxis, including the signs and symptoms of an anaphylactic reaction Information about the risk of a biphasic reaction Information on what to do if an anaphylactic reaction occurs (use the adrenaline injector and call emergency services) A demonstration of the correct use of the adrenaline injector and when to use it Advice about how to avoid the suspected trigger (if known) Information about the need for referral to a specialist allergy service and the referral process Information about patient support groups	Before discharge, offer the patient suitable information regarding the recognition of anaphylaxis signs and symptoms and the correct use of the adrenaline autoinjector	I10, I18, I19
ES9	[19]	After emergency treatment for suspected anaphylaxis, offer people a referral to a specialist allergy service (age-appropriate where possible) consisting of healthcare professionals with the skills and competencies necessary to accurately investigate, diagnose, monitor and provide ongoing management of, and patient education about, suspected anaphylaxis	After emergency treatment of suspected anaphylaxis, refer the patient to a specialist allergy service for future management	I10, I20
ES10	[17]	Referral to an allergist-immunologist is recommended for patients who might be candidates for VIT	As for ES9	
Diagnosis (D)				
D1	[28]	History includes the description of the symptoms and of the course of the reaction (possibly documented by a medical report), the number of stings, the characteristics of the culprit insect (where possible) and the identification of specific risk factors for the severity of reaction	Collect careful clinical history of the reaction, including the possible trigger factors	I1, I12
D2	[28]	Since it is possible to document a sensitization to the venoms in 10–30% of subjects with negative history, only patients with a history of previous systemic reaction should be investigated	Perform diagnostic investigations only in patients with a clinical history of previous systemic reaction	I12, I13
D3	[28]	In patients with a history of LLR, skin tests (as well as specific IgE determination) may be considered as optional, at the discretion of the clinician in specific cases, like in patients at a greater risk of re-sting with recurrent and bothersome LLRs (e.g. beekeepers, farmers) who could benefit from immunotherapy	Perform skin tests in patients with a clinical history of LLR at higher risk of re-sting with the aim of prescribing a possible VIT	I13, I20
D4	[28]	Sting challenge with a live insect should not be used for diagnostic purposes, due to the risk of systemic, potentially severe reactions and low negative predictive value	Do not perform sting challenge for diagnostic purposes	I13
D5	[28]	Allergy to bumble bees, due to its low aggressiveness, concerns a limited number of subjects, in particular professionally exposed individuals [8], and it should therefore be investigated on the basis of a specific anamnestic suspicion, provided that a suitable extract is commercially available for diagnosis	In case of a suspected clinical history, perform diagnostic investigations for Bumble bee also with the aim of prescribing a possible VIT	I12, I13
D6	[28]	In Europe, standardized venoms of Apis mellifera, *Vespula* spp., *Polistes* spp., Vespa crabro are currently, available. The venoms of *Vespula* and *Polistes* consist of a mix of clinically relevant species. Because of low cross-reactivity between European and American Polistes venoms, extracts of *Polistes dominula* are now available for both diagnosis and VIT	Use *Polistes dominula* venom both for diagnostic tests and VIT	I13, I21
D7	[28]	The prick test is carried out at the 100 μg/mL concentration	Perform skin prick tests to the biggest concentration of 100 μg/mL	I13
D8	[28]	Intradermal tests should be performed even in case of positive prick test to identify correctly the cutaneous end-point which will be useful in VIT follow-up	Perform intradermal test even in case of positive skin prick test for diagnosis and VIT follow-up	I13
D9	[28]	Intradermal tests should be carried out by the administration of 0.02 mL of the allergenic extract into the dermis, causing the development of a wheal approximately of 3 mm in diameter. The reading should be performed after 15–20 min; the positivity is documented by an increase of at least 3 mm of the average diameter of the initial wheal, with associated erythema. To allow comparison of results, a morphological score should be used, which consists in drawing, on transparent cellophane, the area injected and the area of the reaction after 15–20 min of time	Perform intradermal test administering 0.02 mL of the allergenic extract, collecting the result after 15–20 min. To allow the comparison of the results, use a morphological score consisting in drawing on a transparent tape the injected area and the reaction area after 15–20 min	I13
D10	[28]	The panel of Italian experts, considering that available data are insufficient, recommends a preliminary step where the same concentration of more venoms is simultaneously used for skin testing. Only after reading the reactions to this first set, a higher concentration should be used. This caution is to be maintained specially in patients with severe anaphylactic reaction or suffering from mast cells disorders	Perform simultaneously the same concentration of different venoms and hereafter the following concentration	I13
D11	[20]	Do not rely on the degree of sensitivity on skin or in vitro testing because it does not reliably predict the severity of a sting reaction	Do not use skin or serological reactivity to predict the reaction severity following the sting	I13, I14
D12	[17]	If initial test results are negative in a patient with a clear history of systemic sting reaction, further testing (in vitro testing, repeat skin testing, or both) should be performed, as well as basal serum tryptase measurement	Perform additional tests (in vitro tests, skin tests repetition, or both, and basal serum Tryptase level) in patients with a clear history of a systemic reaction after a sting and negative skin tests	I13
D13	[22]	If the allergic reaction in mastocytosis patients occurred many years before, the negativity of test could depend from this physiologic decrease and it could be useful to repeat further tests in order to detect a possible sensitization	If the allergic reaction in mastocytosis patients occurred many years before, repeat allergy tests when negative and perform second and third level diagnostic tests	I13, I15, I17
D14	[28]	IgE to CCD can explain multiple in vitro positive results; serum determination for CCD (bromelain or MUXF3) allows greater diagnostic accuracy	Detect serum IgE to CCD (bromelin or MUXF3) in case of multiple in vitro positive results	I13
D15	[28]	Api m 1, the most relevant allergen of bee venom, is not sensitizing in up to 43% of cases. The combination of 2 allergens (Api m 1 and 10) allows diagnosis in 86.8% of cases; the combination of 6 allergens (Api m 1–5, Api m 10) has a sensitivity of 94.4%	Simultaneously detect several bee recombinant allergens (all the disposable allergens on the market) to improve the diagnostic sensitivity	I13
D16	[28]	Patients with *Vespula* spp. venom allergy are sensitized mainly to Ves v 1 and Ves v 5. The combined search of specific IgE toward these two recombinant allergens allows the identification of 92–94% of patients allergic to Vespula	Simultaneously detect specific IgE to Ves v 1 and Ves v 5 in patients with *Vespula* spp. (Yellow jacket) venom allergy	I13
D17	[3]	More recently, some authors used Ves v 1 and Ves v 5 (Immulite system) to diagnose 27 patients with yellow jacket venom allergy and 53 patients with yellow jacket venom allergy and mastocytosis and/or elevated baseline serum tryptase. This study confirmed that the analyses of sIgE reactivity on a component-resolved level revealed no obvious differences in the reactivity profiles of Hymenoptera venom-allergic patients of two groups; in contrast, it showed that a diagnostic sensitivity of 100% was reached in the mastocytosis group using the recombinant allergens an the cutoff of 0.10 kUA/L, instead of the cutoff of 0.35 kUA/L	In patients with *Vespula* spp. (Yellow jacket) allergy suffering or not from mastocytosis and/or elevated basal serum tryptase, use a cut-off of 0.10 kUA/L to dose IgE specific levels to Ves v 1 and Ves v 5	I13, I15, I17
D18	[28]	Another method to distinguish the double sensitization from cross-reactivity is CAP inhibition, although it may be relatively expensive and difficult to interpret. Its use, where available, appears to be very useful in case of double Vespula-Polistes cosensitization, when CRD does not suffice to discriminate the different possibilities	Perform CAP-inhibition in case of double *Vespula*-*Polistes* co-sensitization, when CRD is not discriminating	I13
D19	[28]	The Basophil Activation Test (BAT) is the most widely used in Europe for diagnostic purposes, in selected situations. If performed in highly specialized laboratories, it can identify approximately two-thirds of patients with positive history and negative skin and serological tests	Perform basophil activation test (BAT) in patients with positive clinical history of a systemic reaction and negative skin and serological tests	I13
D20	[28]	BAT is also recommended in patients with double positive results and inconclusive results of in vivo or in vitro tests with recombinant allergens	Perform BAT in patients with double positive results and inconclusive results of in vivo or in vitro tests with recombinant allergens	I13
D21	[3]	In patients with HVA, the BAT was proposed as a third-level test for selected cases, and it can be useful in polysensitization patients	As for D20	
D22	[28]	The role of BAT as a diagnostic tool in patients with mastcell disorders and negative venom-specific IgE and skin test results is still controversial	Use with caution BAT for the diagnosis in patients suffering from mastocytosis with negative allergy tests	I13
D23	[28]	It should be pointed out that patients should be investigated for mastocytosis even in the absence of cutaneous manifestations compatible with mast cell pathology and increased tryptase levels, in case a severe anaphylactic reaction with syncopal episode without urticaria and/or angioedema and a REMA score ≥ 2	Perform mastocytosis diagnostic tests in case of a severe anaphylactic reaction with syncopal episode without cutaneous/mucosal symptoms, with normal basal serum tryptase provided that REMA score is ≥ 2	I15, I17
D24	[17]	Counsel patients with elevated basal serum tryptase about the clinical significance of potential underlying mast cell disorders	Counsel patients with elevated basal serum tryptase level and/or a severe systemic sting reaction about the potential underlying mast cell disorders	I15, I17
D25	[17]	Consider measuring basal serum tryptase in all patients who are candidates for VIT	Measure basal serum tryptase level in all patients who are candidates to VIT	I13, I20
D26	[6]	An anaphylaxis management plan should be used from the time of diagnosis to prevent future reactions, and aid recognition and treatment of any further reactions	Use an anaphylaxis prevention and management plan from the time of diagnosis	I18, I19
D27	[20]	Individualize avoidance measures taking into consideration factors such as the patient’s age, activity, occupation, hobbies, residential conditions, access to medical care, and level of personal anxiety	Personalize the avoidance measures	I18
Pharmacological treatment (TF)				
PT1	[7]	Adrenaline is potentially lifesaving and must therefore promptly be administered as the first-line treatment for the emergency management of anaphylaxis	Administer adrenaline as the first-line anaphylaxis treatment because of its α- and β-adrenergic effect	I19
PT2	[28]	Adrenaline slows the progression of symptoms and can prevent the development of fatal or biphasic reactions	Administer adrenaline as a first-choice anaphylaxis treatment, even for preventing biphasic reactions	I2, I19
PT3	[18]	Epinephrine should be injected by the intramuscular route in the mid-anterolateral thigh as soon as anaphylaxis is diagnosed or strongly suspected, in a dose of 0.01 mg/kg of a 1:1000 (1 mg/mL) solution, to a maximum of 0.5 mg in adults (0.3 mg in children). Depending on the severity of the episode and the response to the initial injection, the dose can be repeated every 5–15 min, as needed	Administer adrenaline by an intramuscular route in the mid-anterolateral thigh in a dose of 0.01 mg/kg of a 1:1.000 (1 mg/mL) solution, to a maximum of 0.5 mg in adults (0.3 mg in children 30 kg of weight). The dose con be repeated every 5–15 min, as needed	I2, I19
PT4	[7]	Aspiration of adrenaline from a vial is time-consuming and the delay may prevent the beneficial effects of the drug; therefore, AAIs are recommended	Prescribe adrenaline in autoinjector device	I19
PT5	[28]	In obese or overweight patients, the reduced length of the needle does not always ensure the intramuscular administration, therefore the patient should be advised to press well the autoinjector on fatty thigh, to compress it and allow the penetration of the needle into the muscle	Instruct obese patients to press well the autoinjector on fatty thigh to compress it and allow the penetration of the needle into the muscle	I19
PT6	[28]	If a correct dosage is administered, it can be used, without absolute contraindications, in pediatric and geriatric populations and in cardiopathic patients, except for some cardiac pathologies such as for example long QT syndrome (in this case, the administration should be performed with extreme caution, in case of real need and in the presence of the cardiologist)	Administer adrenaline in pediatric and geriatric population and in cardiopathic patients in case of anaphylaxis. In patients suffering from some cardiac pathologies, such as long QT syndrome, administer adrenaline in the presence of the cardiologist	I19
PT7	[28]	Adrenaline remains the drug of choice for the treatment of anaphylaxis also for pregnant women	Administer adrenaline to pregnant women in case of secure anaphylaxis	I2, I19
PT8	[20]	Supply any patient who has experienced an episode of anaphylaxis for which the allergen cannot be easily and completely avoided with an AIE and instructions as to when and how to administer this injector and emphasize that they should carry 2 AIEs with them at all times	Any patient with a clinical history of anaphylaxis should carry with him two adrenaline autoinjectors	I19
PT9	[28]	Adrenaline autoinjector should be prescribed to the following categories of patients: children and adults undergoing VIT, but with risk factors for incomplete clinical protection (very severe onset reaction, adverse reactions during immunotherapy, lack of sting protection during VIT, bee venom allergy	Prescribe adrenaline autoinjector to children ad adults undergoing VIT with risk factors for incomplete clinical protection	I19, I21
PT10	[7]	AAI prescription It is also indicated in healthy subjects with a documented anaphylactic sting reaction and negative testing for Venom Specific IgE until second allergy work-up within 6 weeks to 3 months later is not performed	Prescribe adrenalin autoinjector in patients with documented anaphylactic sting reaction and negative specific IgE until second allergy work-up	I10, I19
PT11	[6]	Underlying mast cell disorders or elevated baseline serum tryptase concentrations together with any previous systemic allergic reactions to insect stings, even in VIT-treated patients are an absolute indications for prescription at least one adrenaline auto-injector	Prescribe at least one adrenaline autoinjector in patients with mast cell disorders or elevated baseline serum tryptase concentrations and with history of previous sting systemic reaction, even if in VIT treatment	I16, I19
PT12	[7]	The prescription of a second AAI is indicated in patients with mast cell diseases and/or raised BST, previous requirement for more than one dose of adrenaline prior to reaching hospital, previous near-fatal anaphylaxis, lack of rapid access to medical assistance to manage an episode of anaphylaxis due to geographical or language barriers	Prescribe a second adrenaline autoinjector in patients with risk factors for a severe reaction	I16, I19
PT13	[7]	The prescription of an AAI is indicated in patients with underlying mast cell disorders and anaphylactic sting reactions and negative testing for venom-specific IgE	Prescribe adrenaline autoinjector in patients with mast cell disorders and clinical history of anaphylactic reaction even if with negative IgE specific tests	I16, I19
PT14	[28]	Patients affected by SM with a history of anaphylaxis should always carry two adrenaline autoinjectors, a recommendation also valid for patients receiving VIT	Prescribe two adrenalin autoinjectors in patients affected by systemic mastocytosis and with history of anaphylaxis, even if receiving VIT	I16, I19
PT15	[3]	These patients should carry a self-administration emergency kit that includes oral antihistamines and corticosteroids as well as self-injectable epinephrine	Prescribe to patients with systemic mastocytosis: adrenaline autoinjectors, oral corticosteroids and antihistamines	I16, I19
PT16	[28]	Adrenaline autoinjector should be prescribed to the following categories of patients: children and adults with systemic reactions more severe than systemic skin reaction or with a high risk of re-exposure to sting (e.g. beekeepers), before VIT	Prescribe adrenaline autoinjector to children and adults with systemic reactions or with high risk of re-exposure to sting	I19
PT17	[7]	AAI prescription should be considered in patients with previous mild (cutaneous) sting reaction and remote from medical help	Take into consideration the prescription of an adrenaline autoinjector in patients with previous cutaneous reaction and remote from an Emergency Department	I19
PT18	[8]	During and after VIT, AAI cannot be recommended in patients with mild-to-moderate initial sting reactions without risk factors for relapse	Do not prescribe adrenaline autoinjector to patients with mild to moderate initial sting reaction without risk factors for relapse if they are under VIT or have completed VIT	I19, I20
PT19	[28]	The Italian experts do not rule out the possibility of prescribing adrenaline to patients at risk of multiple stings (e.g. beekeepers) and to those who have developed a single LLR, since in these subjects the risk of a subsequent systemic reaction to a re-sting cannot be completely excluded compared to patients who have already shown repeated LLR	Prescribe adrenaline autoinjector to patients with large local reactions when at risk of multiple sting (e.g. beekeepers) and to those who have developed a single LLR	I19
PT20	[7]	Patients experiencing anaphylaxis should be advised on other interventions needed to manage the reaction. They should be advised to call for help, if possible, and adjust their position according to their leading symptoms: When respiratory distress is leading, they should sit or remain seated, and when symptoms of circulatory instability are leading, they should lie down on their back with the lower extremities elevated	Advise patients with history of anaphylaxis on the interventions needed to manage the reaction, in particular how to adjust their position	I18
PT21	[17]	Advise the patient to treat acute systemic reactions to insect stings like any anaphylactic reaction, with timely transport to an Emergency Department	Treat acute systemic reactions to sting like potential anaphylactic reactions, with timely transport to an Emergency Department	I1, I2, I18
PT22	[16]	Do not routinely administer antihistamines or corticosteroids instead of epinephrine. There is no substitute for epinephrine in the treatment of anaphylaxis Administration of H1 and/or H2 antihistamines and corticosteroids should be considered adjunctive therapy	Do not administer antihistamines or corticosteroids instead of adrenaline during an anaphylactic reaction, but only as a second line adjunctive therapy	I2, I19
PT23	[7]	Glucocorticoids in the treatment of anaphylaxis potentially relieve protracted anaphylaxis symptoms and are thought to prevent biphasic anaphylaxis, although these effects have never been proven	Administer corticosteroids to relieve protracted anaphylactic symptoms (however not in adrenaline replacement) and probably to prevent biphasic anaphylaxis, although this effect has never been proven with controlled randomized trials	I2
PT24	[7]	Patients may receive a set of tablets containing an adequate dose of a rapidly effective non-sedating oral antihistamine (e.g. levocetirizine 10 mg, cetirizine 20 mg, or double dose for children according to the age) and corticosteroids (e.g. prednisone: for adults 50–100 mg and 1–2 mg/kg body weight in children). For mild SARs, oral antihistamines and corticosteroids are a sufficient treatment. However, their use should not delay self-treatment with an AAI if one is carried and extracutaneous symptoms occur	Prescribe oral antihistamines and corticosteroids only for mild systemic reactions	I2
PT25	[17]	Treat large local reactions symptomatically, with antihistamines, cold compresses, and analgesics as needed. In severe cases a short course of oral corticosteroids may be useful. Antibiotics are usually not necessary and should be prescribed only if specifically indicated	Treat large local reactions with cold compresses, eventually with pharmacological therapy based on oral antihistamines and analgesics. In severe cases administer oral corticosteroids. Antibiotics are usually not necessary	I18
PT26	[7]	Patients with asthma should be advised to carry their inhaled short-acting beta-2-agonist and to use as many inhalations as needed if respiratory difficulty follows a sting	Advise patients with asthma to use inhaled short-acting β2-agonist if respiratory difficulty follows a sting	I18
Venom immunotherapy (VIT)				
VIT1	[17]	Begin VIT with initial dose of up to 1 µg and increase to maintenance dose of at least 100 µg of each venom	Begin VIT with initial dose of up to 1 µg and increase to maintenance dose of at least 100 µg of each venom	I21
VIT2	[8]	It is recommended to administer a standard maintenance dose of 100 µg venom	Administer a standard maintenance dose of 100 µg venom	I21
VIT3	[8]	Purified venom preparations can be recommended as they have a lower frequency of local and systemic adverse events than non-purified aqueous preparations	Recommend aqueous purified venom preparations rather than non-purified aqueous venom preparations as they are better tolerated and have lower risk of systemic reactions during VIT	I21
VIT4	[8]	It may be recommended that patients treated with bee venom and those on rapid up dosing protocols should be closely observed for side-effects as they are at a higher risk of experiencing adverse events	Closely observe patients under VIT with bee venom and those on rash or ultra-rash up-dosing protocols	I21
VIT5	[8]	In case of VIT-related systemic adverse events during build-up phase, a temporary reduction of the venom dose (e.g. going one to two steps back in the protocol) may be recommended to avoid further adverse events	In case of VIT-related systemic reactions during build-up phase, temporarily reduce the venom dose of one to two steps in the VIT protocol	I21
VIT6	[8]	It may be recommended to avoid insect stings during build-up phase by abiding by preventive measures (e.g. stop beekeeping) until maintenance dose is reached	Recommend avoiding hymenoptera stings during build-up phase by abiding preventive measures (e.g. stop beekeeping) until maintenance dose is reached	I18, I21, I22
VIT7	[8]	In case of repeated systemic adverse events during up dosing, pretreatment with Omalizumab may be recommended	Pretreat with Omalizumab in case of repeated systemic adverse events during VIT up-dosing, taking into account it is an “off-label” therapy	I21
VIT8	[3]	Several case reports showed that pretreatment with anti-IgE monoclonal antibodies may permit more rapid and higher doses of allergen immunotherapy: patients with ISM who experienced SRs to VIT were able to tolerate immunotherapy after pretreatment with Omalizumab	Perform “off label” treatment with Omalizumab in patients with mastocytosis who experienced systemic reactions during VIT	I21, I16
VIT9	[8]	If patients still react to field stings or sting challenges, a dose increase to 200 µg of venom can be recommended	Increase venom maintenance dose to 200 µg if patient is not protected with the dose of 100 µg by a field sting	I21
VIT10	[3]	In patients with HVA and SM not fully protected at field re-stings, an increase of the maintenance dose to 200 μg of venom could be recommended	Increase venom maintenance dose to 200 µg in patients with systemic mastocytosis not fully protect by standard dose of 100 µg	I21
VIT11	[17]	Treatment with some venoms may not be needed if cross-reactivity can be demonstrated by a radioallergosorbent inhibition test	Treatment with different venoms is not needed if cross-reactivity can be demonstrated	I13, I21
VIT12	[28]	VIT is indicated in the following circumstances: (a) Children and adults with a systemic reaction involving other apparatus besides the skin. (b) Systemic skin reactions at high risk of exposition and/or impairment of quality of life in adults. (c) Patients with clonal mast cell disorder and a history of a systemic reaction, even though sensitization can be weak or sometimes transitory	VIT is indicated in: (i) adults and children with a systemic reaction involving other apparatus besides the skin; (ii) systemic skin reaction at high risk of exposure and/or impairment of quality of life; (iii) patients with clonal mast cell disorders and a history of a systemic reaction, even though sensitization can be sometimes transitory	I20
VIT13	[28]	In children with only cutaneous systemic reactions, VIT is not routinely performed. However, there may be particular situations of increased risk of re-sting (e.g. children of beekeepers), possibly associated with parents’ and children’s concern or with distance from the emergency room, or unavailability of school staff administering antiallergic drugs. These conditions warrant VIT also in cases of urticarial alone	Consider to perform VIT in children with only cutaneous systemic reactions if there are particular situations (e.g. increase risk of re-sting, concern, or distance from the Emergency Department, etc.)	I20
VIT14	[17]	In a change from previous recommendations, advise both children and adults who have experienced only cutaneous systemic reactions without other systemic manifestations after an insect sting that VIT is generally not required but may be considered when there are special circumstances. This should be a shared decision with consideration of high-risk factors (frequent exposure, cardiovascular or respiratory conditions, or selected medications) and the effects on quality of life	Advise both children (and caregivers) and adults who have experienced only cutaneous systemic reactions after an insect sting that VIT is generally not required. VIT can be considered when there are special circumstances (cardiovascular or respiratory conditions, effects on quality of life)	I20
VIT15	[28]	In patients allergic to Hymenoptera venom, in whom a subsequent allergic reaction may be more severe or even fatal, VIT has an elective indication even if there has been a myocardial infarction or a severe ventricular arrhythmia	Suggest VIT in cardiopathic venom allergic patients when a systemic reaction occurred	I20, I21
VIT16	[28]	VIT should be taken into consideration in older adults, even if they have experienced a non-severe systemic reaction, provided that they have risk factors such as: concomitant vascular diseases, treatment with ACE inhibitors and/or beta-blockers, severe COPD pictures, reduced quality of life due to the previous anaphylactic event	Suggest VIT in older adults, even in case of non-severe systemic reaction, when they present risk factors for severe reactions at re-sting, such as vascular diseases, treatment with ACE-inhibitors and/or β-blockers, severe COPD	I1, I12, I20
VIT17	[8]	For the majority of patients, VIT with one venom may be recommended as sufficient for protection. In patients with a history of systemic sting reactions to different insects or with severe initial reactions and clearly double positive tests, VIT with two venoms (i.e. *Apis mellifera* and *Vespula* or *Vespula* and *Polistes*) is recommended	Suggest VIT with two venoms in patients with a history of systemic sting reaction by not-identified insect or with severe initial reaction and double positive diagnostic tests, in the absence of second and third level diagnostic tests	I20, I21
VIT18	[8]	VIT can be recommended in adults with recurrent, troublesome LLR to reduce the duration and size of future LLR	Take into consideration VIT in adults with recurrent, troublesome late local reactions	I20, I22
VIT19	[8]	VIT is not recommended in individuals with incidentally detected sensitization to insect venom and no clinical symptoms	Do not recommend VIT in individuals with Hymenoptera venom sensitization and no clinical symptoms (asymptomatic sensitization)	I22
VIT20	[8]	VIT may be recommended in patients with underlying systemic mastocytosis as it is safe and effective	Recommend VIT in patients with Hymenoptera venom allergy with underlying systemic mastocytosis	I15, I17, I20
VIT21	[26]	VIT in mastocytosis patients should be performed by experienced allergy centers due to the risk of severe side-effects, including anaphylaxis, and the need for dosage adjustments and concomitant treatments	In patients with systemic mastocytosis perform VIT in experienced allergy centers	I21
VIT22	[28]	Status of cardiovascular disease, its pharmacological treatment and the risk of anaphylaxis with consequent adrenaline administration should be carefully evaluated on an individual basis, preferably in concert with the consulting cardiologist, before starting VIT	Evaluate on individual basis, preferably with the consulting cardiologist, the status of the cardiovascular disease and the pharmacological treatment (especially β-blockers or ACE-inhibitors) before starting VIT for the possible future use of adrenaline	I19, I20, I22
VIT23	[8]	VIT can be recommended in patients with cardiovascular disease but the underlying disease should be stabilized before initiation	Stabilize an underlying cardiovascular disease before starting VIT	I20
VIT24	[8]	Beta-blocker therapy may be continued during VIT but the patient should be informed about possible risks	Inform the patients about the possible risks of a beta-blocker therapy	I20, I21, I22
VIT25	[8]	ACE inhibitor therapy may be continued during VIT but the patient should be informed about possible risks	Inform the patients about the possible risks of ACE-inhibitor therapy during VIT, particularly about the possible reduced efficacy of VIT	I20, I21, I22
VIT26	[8]	Treatment with MAOIs is not a contraindication for VIT but caution is recommended with the use of adrenaline	Recommend caution in using adrenaline during VIT in patients in MAO-inhibitory treatment	I20, I19
VIT27	[28]	In patients allergic to Hymenoptera venom with a high risk of severe reactions to subsequent stings (e.g. previous life-threatening reaction or clonal mast cell diseases), VIT appears to prevent fatal events even in the presence of neoplasia	Suggest VIT to patients with neoplasia, stable or in remission, and high risk of severe reactions to subsequent sting or clonal mast cell diseases	I20
VIT28	[28]	VIT is not generally indicated in unusual reactions (i.e. serum-like sickness manifestations, manifestations of central nervous system, hematological, muscle and renal reactions)	Do not prescribe VIT in unusual reactions, not IgE-mediated	I20
VIT29	[28]	Immunotherapy should not be started during pregnancy. However, VIT should not be interrupted in case of pregnancy if patients are already undertaking and well tolerating VIT, considering the low risk of side effects	Do not start VIT during pregnancy, however a well tolerated VIT should not be interrupted in case of pregnancy	I20, I21
VIT30	[28]	VIT cannot be recommended in patients with active, multisystem autoimmune disorders	Do not administer VIT in patients with active multisystem autoimmune disorders	I20
VIT31	[28]	VIT is not contraindicated in patients with organ-specific autoimmune diseases (e.g. diabetes mellitus, Hashimoto’s thyroiditis, Crohn’s disease, ulcerative colitis, rheumatoid arthritis), provided the disease is stabilized before starting treatment	Start VIT in patients with organ-specific autoimmune disease when the disease is stabilized	I20
VIT32	[28]	HIV infection is a relative contraindication to VIT that can be assessed on an individual basis	Assess VIT on an individual basis in patients with HIV infection	I20
VIT33	[28]	AIDS with a confirmed category C disease (according to CDC 1993 Atlanta Classification) is an absolute contraindication to VIT	Do not perform VIT in patients with AIDS with a confirmed category C disease (according to CDC 1993)	I20
VIT34	[28]	In patients without specific risk factors VIT should be continued for 5 years	In patients without risk factors VIT should be continued for 5 years	I20, I21
VIT35	[8]	It may be recommended to give injections every 4 weeks in the first year of treatment, every 6 weeks in the second year, and in case of a 5 year treatment every 8 weeks from year 3–5	Administer VIT every 4 weeks in the first year of treatment, every 6 weeks in the second year, and every 8 weeks from year 3 to 5	I21, I22
VIT36	[8]	In the case of lifelong therapy, 12-week intervals may be still safe and effective	In case of a VIT prolonged over 5 years a progressively increased maintenance interval till to 12 weeks con be applied	I21
VIT37	[28]	The panel of experts suggests that VIT duration should be at least 5 years also in pediatric patients	Suggest a VIT duration of 5 years in pediatric patients	I20, I21
VIT38	[17]	Encourage continuation of VIT for an extended time, or indefinitely, in patients with high-risk factors, such as very severe reaction before VIT (syncope, hypotension, severe respiratory distress), systemic reaction during VIT, honeybee allergy, and increased basal serum tryptase levels	Encourage VIT for an extended time (more than 5 years) or indefinitely in patients with high risk factors for relapse	I21, I22
VIT39	[17]	Consider continuation of VIT for more than 5 years in patients with other high-risk factors for recurrent or severe sting reactions, such as underlying cardiovascular or respiratory conditions, select antihypertensive medications, frequent exposure, and limitation of activity due to anxiety about unexpected stings	As for VIT38	
VIT40	[26]	Patients with IgE-mediated anaphylaxis to Hymenoptera should be offered venom immunotherapy (VIT) to honeybee or wasp or both and should be performed life-long in patients with mastocytosis	Suggest life-long VIT in patients with mastocytosis	I20, I16
VIT41	[28]	Since workers highly exposed to stings have a higher risk of relapse after VIT discontinuation, some experts recommend to continue the treatment until the profession risk is maintained	Continue VIT until the profession at risk of frequent stings is maintained	I20, I21, I22
VIT42	[8]	Lifelong VIT may be recommended in patients who relapsed after stopping VIT	Start again and continue lifelong VIT in patients who relapsed after stopping VIT	I21
VIT43	[3]	From a practical point of view, regardless of the tryptase value, we would suggest an accurate hematologic workup be performed before stopping immunotherapy in those patients with very severe reactions with hypotension and without urticaria and angioedema to exclude CMD	Before stopping VIT in patients with very severe reactions with hypotension and without urticaria/angioedema, perform an accurate hematologic workup to exclude clonal mast cell disorders	I15, I17, I21, I22
VIT44	[21]	Patients with HVA-induced anaphylaxis who lose protection after a proper course of VIT should be investigated for mastocytosis	Perform an accurate hematologic workup to exclude clonal mast cell disorders in patients with Hymenoptera venom allergy who lost protection after stopping VIT	I15, I17, I22
VIT45	[8]	If no sting challenge can be performed, it may be recommended to record outcomes of field stings to evaluate effectiveness of VIT	Record outcomes of field stings to evaluate effectiveness of VIT	I21, I22
VIT46	[8]	It may not be recommended to determine venom specific IgE, IgG levels, BAT response and allergen blocking capacity to estimate the individual risk for relapse	Do not use specific IgE and IgG levels, BAT response and CAP inhibition to estimate the individual risk for relapse	I13, I22
VIT47	[28]	Patients at risk of multiple stings or with risk factors for relapse after VIT interruption: follow up visit in case of re-sting and anamnestic history collection at each re-order of adrenaline including a refresh in the training on device use	After stopping VIT, perform follow-up visit in case of re-sting, collecting clinical history especially in patients at risk for multiple stings or with risk factors for relapse; at each re-order of adrenaline include a refresh in the training on device use	I22
Mastocytosis (M)				
M1	[27]	The European Competence Network on Mastocytosis recommends using the REMA score as a clinically useful tool to predict for the presence of clonal MCs prior to a BM study; the REMA score is based only on demographic data (gender), the symptoms and signs observed during the acute episodes, and serum baseline tryptase levels. A REMA score of at least 2 predicts with a high sensitivity and specificity for ISMs- (or c-MCAS), whereas a REMA score of less than 2 usually indicates non-clonal disease. The REMA score is a particularly helpful tool since it is based on clinical data and can be used on a routine clinical basis, it is associated with rather low costs, and it avoids unnecessary BM studies	Use REMA score to predict a clonal mast cell disease before submitting the patients to bone marrow biopsy	I15, I17
M2	[24]	Subjects with anaphylaxis after wasp or bee stings and negative allergy test results might have unrecognized mastocytosis. In these subjects, investigations for major and minor SM criteria are recommended, regardless of Serum baseline tryptase (sBT) level or the presence of skin lesions	Perform an accurate hematologic workup to detect major and minor criteria of systemic mastocytosis in patients with Hymenoptera venom anaphylaxis and negative diagnostic tests	I13, I15, I17
M3	[21]	In cases with a clearly raised SBT level (> 25 ng/mL), BM biopsy is usually recommended, whereas in patients with systemic HVA reactions and borderline or normal tryptase, the scoring system proposed by REMA (score) can be useful to decide whether patients should undergo BM biopsy	Perform a bone marrow biopsy in case of clearly raised serum basal tryptase (> 25 ng/mL). In patients with systemic Hymenoptera venom reaction and borderline or normal serum basal tryptase level, use REMA score to decide whether patients should undergo bone marrow biopsy	I15, I17
M4	[24]	In the presence of a very low BM MC burden, KIT D816V mutation analysis should be performed with very sensitive techniques, such as quantitative RT-PCR	Diagnose systemic mastocytosis performing KIT D816VT mutation analysis with a very sensitive technique, such as RT-PCR, in the presence of a very low bone marrow mast cell burden (major criterion)	I15, I17
M5	[24]	Assessment of sBT levels is an inexpensive, reliable, and simple screening test for mastocytosis in subjects with a positive history of a systemic reaction to hymenoptera stings	Determine basal serum tryptase level in all the patients with positive history of hymenoptera sting systemic reaction	I13, I15, I17
M6	[26]	In the follow-up and monitoring of patients with mastocytosis s-tryptase is a widely used marker of MC burden	Use serum basal tryptase levels as a marker of mast cells burden to follow-up patients with mastocytosis	I22
M7	[3]	In these patients, it is very important to use a highly sensitive multiparameter flow cytometry approach to stain BM cells, using a combination of five monoclonal antibodies—CD45, CD117, CD34, CD25, and CD2—and with at least 1 to 3 million events acquired to detect atypical MC in these patients who have a very low MC burden	In patients with systemic mastocytosis, use a highly sensitive multiparameter flow cytometry with a combination of five monoclonal antibodies (CD45, CD117, CD34, CD25 e CD2)	I15, I17
M8	[22]	It is important to confirm the diagnosis of c-MCAS in patients with HVA: (i) before to decide to stop VIT because, in patients with a diagnosis of c-MCAS, VIT must be continued lifelong. (ii) in order to give them a proper advice on the use of adrenaline. (iii) to investigate and manage other manifestations of c-MCAS, such as osteoporosis	Confirm the diagnosis of c-MCAS in Hymenoptera venom allergic patients to decide VIT duration, adrenaline autoinjector prescription as well as the evaluation of other clinical manifestation associated to systemic mastocytosis	I15, I16, I17, I19, I21
M9	[26]	Of note, s-tryptase is not disease-specific for mastocytosis and may be elevated in healthy individuals, and in other conditions, such as chronic urticaria, kidney failure, chronic helminth infections, or other myeloid haematological diseases A similar, step-wise approach, including KIT D816V testing in the algorithm is recommended by the ECNM (European Competence Network on Mastocytosis)	Diagnose systemic mastocytosis with other tests in addition to basal serum tryptase among which KIT D816VT mutation analysis	I15, I17
M10	[26]	In general, these centres recommend and perform a thorough BM examination in order to exclude or to establish the diagnosis of SM, to assess the BM MC burden, and to rule out or demonstrate the presence of another (associated) hematological disease	Perform a thorough bone marrow biopsy to exclude or confirm the diagnosis of systemic mastocytosis, to evaluate the mast cells burden, and to exclude or detect another hematological associated disease	I15, I17
M11	[22]	The diagnosis of SM is made upon the presence of the major criterion (histological finding of at least 15 multifocal dense MC infiltrates in BM or other extracutaneous organs) plus 1 minor criterion, or at least 3 out of 4 minor criteria (abnormal morphology of extracutaneous MC; serum tryptase > 20 ng/m; expression of CD2 and/or CD25 on BM MC; detection of a mutation at codon 816 of the KIT gene in extracutaneous organs). Clinical features associated with MC burden (B findings) or aggressiveness of disease (C findings) are also applied to subclassify patients with SM	Diagnose systemic mastocytosis upon the presence of a major plus a minor criterion, or at least three minor criteria. Subclassify systemic mastocytosis in indolent, smoldering or aggressive on the basis of the presence of B and/or C findings presence	I15, I17
M12	[22]	MCAS can be diagnosed when patients have recurrent systemic, usually severe, symptoms of MC activation, and involvement of MC can be documented preferably by demonstrating a transient increase of serum tryptase or another established MC mediator during (or shortly after) an event; moreover, symptoms need to respond to anti-mediator-type or MC-stabilizing medications. All these criteria should be fulfilled in order to confirm the diagnosis of MCAS	To confirm the MCAS diagnosis, all the established criteria need to be satisfied: recurrent systemic symptoms responsive to anti-mediator therapy or to mast cell stabilizing medications, transient increase of serum tryptase or another established mast cell mediator during an event	I15, I16, I17
M13	[26]	In children the diagnosis of CM is largely clinical and a BM examination is not warranted or recommended except for the very rare situation where the child does not thrive and, in addition, shows persistently high and rising s-tryptase levels, abnormal blood count, or hepato/splenomegaly	Diagnose cutaneous mastocytosis in the children by clinical examination; bone marrow biopsy is not warrant nor recommended except for very rare situations.	I15, I17
M14	[26]	In all adult patients and all childhood patients, a precise and complete physical examination is standard	When suspect systemic mastocytosis carry out a complete physical examination in all the patients, both children and adults	I12, I15, I17
M15	[26]	Elevated baseline serum tryptase is characteristically found in SM, although a normal serum tryptase level does not rule out the presence of mastocytosis	Do not exclude the diagnosis of systemic mastocytosis on the basis of normal basal serum tryptase levels	I15, I17
M16	[24]	The characteristics of severe HVA episodes with hypotension in the absence of urticaria/angioedema might represent the most relevant factor to identify those patients with HVA and CMDs, regardless of baseline tryptase levels. In such patients, who are indeed very rare, a proper and adequate diagnosis should be offered without delay	Perform an accurate diagnostic workup for mastocytosis in patients with a history of severe anaphylaxis and absence of urticaria/angioedema; furthermore do not exclude systemic mastocytosis on the basis of normal basal serum tryptase levels	I15, I16, I17

**Fig. 2:**
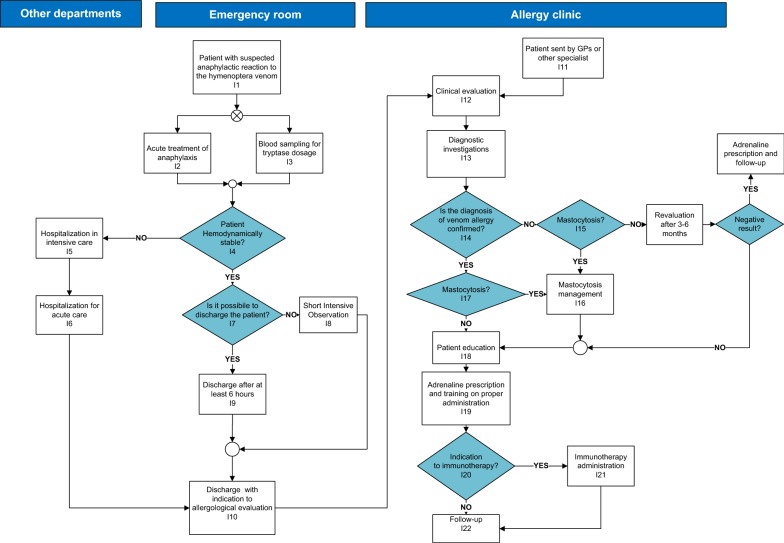
Process flow diagram for the management of HVA patients

The key interventions were presented by means of a process flow diagram (Fig. 2). All the flow chart forms were numbered in order to link them to the recommendations and related key interventions.

Six process indicators were produced by the recommendations that had obtained an average score higher than 8.8 (Table 2).

**Table 2:** Process and outcomes indicators for the management of HVA patients

Number	Indicator	Type	Unit	Value (%)
1	% of patients with severe anaphylaxis receiving acute adrenaline treatment in emergency room (ER)	Process	Percent	90
2	% of patients discharged from ER. with allergological evaluation indication	Process	Percent	90
3	% of patients prescribed a second adrenaline auto-injector if clinically indicated (patients with mast cell diseases and/or with increased baseline serum tryptase levels, previous need for more than one dose of adrenaline before reaching the hospital, almost fatal previous anaphylaxis, lack of rapid access to anaphylaxis medical assistance due to geographical or linguistic barriers, patient’s habitus)	Process	Percent	90
4	% of patients with multiple positivity in whom complete CRD was performed	Process	Percent	95
5	% of patients with severe anaphylactic reaction with syncopal episode without urticaria and/or angioedema and a REMA score ≥ 2 initiated for mastocytosis diagnostic investigations	Process	Percent	80
6	% of patients with Hymenoptera venom allergy, myocardial infarction or severe ventricular arrhythmia undergoing VIT	Process	Percent	90

*ET* emergency treatment, *D* diagnosis, *PT* pharmacological therapy, *VIT* venom immunotherapy, *M* mastocytosis

**Fig. 3:**
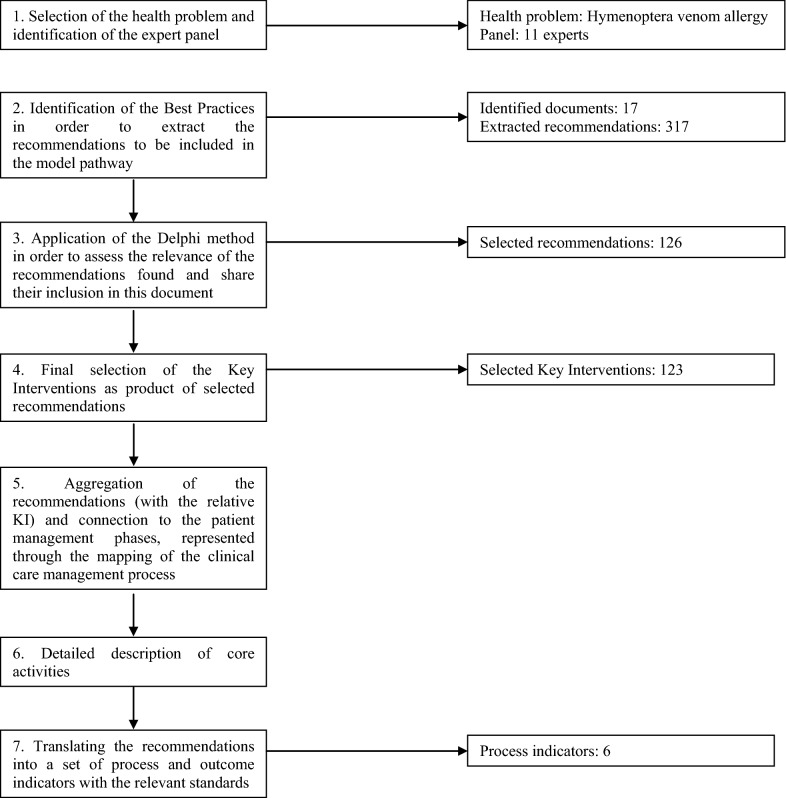
Methodology for the development of the clinical pathway

Figure 3 summarizes the development of the clinical pathway and its results.

Finally, the document was submitted to a panel of other 17 specialists responsible for Allergy Departments in Italy, who did not produce any additional remarks, in particular about KI and indicators. The core activities will then be included within Operative pathways, which are the real representation of routes at the local level, based on resources and available skills. The final document was then shared with the Italian Federation of patient associations (FederAsma e Allergie onlus). Specific questions from patient’s point of view asked by the Italian Federation found their answer in different parts of the document (KI and flow-chart).

## Discussion

Hymenoptera venom allergy is an important cause of morbidity worldwide and may occur with varying degrees of severity and can sometimes be fatal.

The management of the patients who have developed a SR after a Hymenoptera sting provides for a correct management in the emergency treatment, followed by a correct diagnosis that forms the basis for the treatment, represented by the prescription of an adrenaline autoinjector, and VIT, where necessary.

CPWs are used to improve quality of care, standardize care and maximize the outcomes for specific groups of patients [11]. A CPW with the goal to increase the level of medical awareness on HVA and outline clear care paths for these patients is lacking. Therefore, this document aims to identify a shared, multidisciplinary path, based on the most recent national and international scientific evidence (quality guidelines were used for the most part) and supported by the consensus of different specialists with extensive experience in emergency treatment (2) and clinical expertise and scientific knowledge of HVA (7). The multidisciplinary expert group was fundamental for the development of the CPW, making it possible to deal comprehensively with the management of the HVA patients.

The main objective is to standardize the behavior of professionals with regard to taking care, diagnosis, treatment and follow-up of allergic patients with systemic reactions due to Hymenoptera stings, so as to ensure the maximum degree of appropriateness of the interventions and health services and minimizing the degree of variability in clinical decisions.

Professional uncertainty and the scarce use of medical evidence seem to be the key elements in many problems dealing with health care variations, due to their possible links with medical errors. Reducing variations by standardizing clinical processes is an effective tool to minimize the probability of medical errors [29].

Given that HVA is the most frequent cause of severe allergic reactions, and still an epidemiologically underestimated condition, this codified approach also allows a better assessment of the impact of the disease and its epidemiological weight.

Moreover, we developed a set of indicators, which are potentially important tools able to help clinicians and healthcare organizations to assess the quality of care being provided by agreed evidence-based recommendations. Therefore, the selected indicators are relevant for research on quality of management of HVA patients. These indicators should be embedded in daily clinical practice to encourage continuous quality assessment and improvement of care for Hymenoptera venom allergy patients. Six process indicators were selected. Process indicators measure the clinicians compliance to the key interventions in the care process. Key interventions are those that, based on evidence-based medicine, need to be performed to guarantee high-quality care, and that thus will have significant impact on patient outcomes.

It is of note that the document represents the first European HVA CPW produced according to a rigorous methodology. The CPW was made by an allergy expert panel and was submitted to other 17 experts, who approved all the steps, including the KI, the process flow diagram, and the indicators, judged valid and applicable in clinical practice.

It is noteworthy that CPW benefited from the endorsement of three scientific societies (Italian Association of Hospital and Territorial Allergists and Immunologists, AAIITO—Italian Society of Allergology and Clinical Immunology, SIAAIC—Italian Society of Pediatric Allergy and Immunology, SIAIP) which plays an important role in clinical content development for care pathway, especially in terms of clinical support, expert networking and input of resources.

One limitation of the CPW process is the lack of patient involvement since the beginning of the document development. Patients can bring a different perspective to the quality improvement process, as they are likely to prioritize different aspect of care compared to clinicians. However, the definitive document was submitted to the Italian Federation of patient associations, which evaluated and shared the CPW.

Furthermore, we did not verify the indicators in specific clinical audits, which allows to obtain further information on the feasibility of data collection, on verification of adherence to the CPW and on any changes to effectively implement the path; however, this phase was not included in the project.

The next step of project is to translate and check the feasibility of the CPW at local Italian levels. This phase is particularly important as designing the care pathway content is a time-consuming process that requires resources and skills.

## Conclusion

Developing the clinical care pathway may facilitate adequate integration of evidence-based knowledge into daily practice. For the first time in the field of HVA, by using the Delphi survey with 11 experts 123 key interventions were found to be appropriate for the development and standardization of the clinical content of the HVA care pathway. Next to the model pathway, six indicators were identified for monitoring and following up of Hymenoptera venom allergy patients.

Compliance to care pathways can be difficult, but has to be strived for, to deliver the best possible care for patients.

## Data Availability

Not applicable.

## Abbreviations

CRD: Component-Resolved-Diagnosis
VIT: Venom immunotherapy
HVA: Hymenoptera venom allergy
CPWs: Clinical pathways
GPP: Good Practice Point
KI: Key interventions
ET: Emergency treatment
D: Diagnosis of HVA
PT: Pharmacological therapy
M: Mastocytosis and Hymenoptera venom allergy
LGs: Guidelines
SM: Systemic mastocytosis
SR: Systemic allergic reaction
MC: Mast cell
